# Resilience following bereavement: a longitudinal network analysis of depressive symptoms and cognitive functions in older adults

**DOI:** 10.1192/j.eurpsy.2026.11565

**Published:** 2026-07-31

**Authors:** M. Semkovska

**Affiliations:** Department of Psychology, University of Southern Denmark, Odense, Denmark

## Abstract

**Introduction:**

A well-established depression risk factor, bereavement is a major stressful life event (SLE), whose prevalence increases as people age. Depression in later life can signal dementia, while cognitive deficits increase vulnerability to depression. However, how SLEs – particularly bereavement – intervene in the reciprocal relationship between symptoms and cognitive function to trigger clinical depression remains unclear. Clarifying this mechanism is critical, given population aging and the related increase in dementia, for which depression remains a significant predictor. According to network theory, depressive symptoms and related risk or protective factors (e.g. cognitive functioning, SLEs) form an interconnected system in which the strength of associations among components reveals each factor’s specific role in activating the broader network.

**Objectives:**

Network modelling can clarify how interactions between individual depressive symptoms and cognitive functions change over time in bereaved older adults, helping to explain why some show resilience following the loss of a loved one while others develop depression.

**Methods:**

Contemporaneous and cross-lagged panel networks examined cross-sectional and longitudinal associations between depressive symptoms and cognitive functions in 1992 recently bereaved Danish adults aged over 70. Inclusion criteria were to have: (1) recently lost a close relative or a friend, (2) never been depressed before the bereavement occurrence, and (3) complete depressive symptom and cognitive function data at both baseline and a two-year follow-up. The networks of individuals who became depressed at the follow up (n=222) were compared to those who remained resilient to depression (n=1770).

**Results:**

Over the two-year post-bereavement, the resilient networks showed significant structural changes between baseline and follow-up, whereas the depression-onset group maintained a stable network (Figure 1). Significant, between-group differences were observed in the predictions linking depressive symptoms and cognitive functions across time. The resilient group showed few longitudinal predictions. In the depression-onset group, baseline auditory attention, working memory, verbal learning, and delayed recall predicted follow-up depressive symptoms, while follow-up verbal fluency was predicted by baseline depressive symptoms.

**Image 1:**
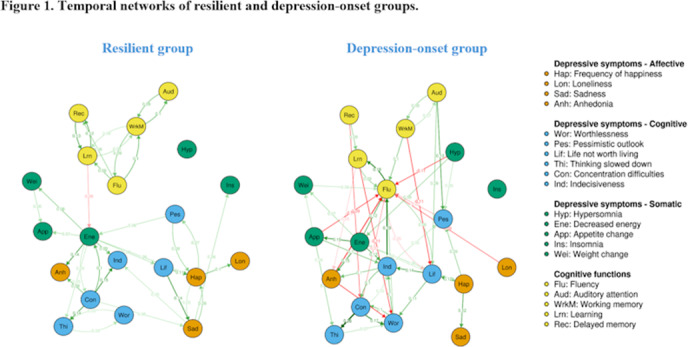
Image 1: Long description.

**Conclusions:**

Following bereavement, resilient older adults showed a flexible extended network of depressive symptoms and cognitive functions. Lower baseline cognitive function within a structurally rigid, self-perpetuating network appeared to trigger depression-onset.

**Disclosure of Interest:**

None Declared

## Long descriptions

### Image 1: Long description

Two network diagrams show nodes representing symptoms and functions connected by green positive or red negative arrows. A legend on the right categorizes nodes into four groups.

* Depressive symptoms - Affective (Orange): Hap (Frequency of happiness), Lon (Loneliness), Sad (Sadness), Anh (Anhedonia).

* Depressive symptoms - Cognitive (Blue): Wor (Worthlessness), Pes (Pessimistic outlook), Lif (Life not worth living), Thi (Thinking slowed down), Con (Concentration difficulties), Ind (Indecisiveness).

* Depressive symptoms - Somatic (Green): Hyp (Hypersomnia), Ene (Decreased energy), App (Appetite change), Ins (Insomnia), Wei (Weight change).

* Cognitive functions (Yellow): Flu (Fluency), Aud (Auditory attention), Wrk M (Working memory), Lrn (Learning), Rec (Delayed memory).

In the Resilient group network on the left, connections are sparse and primarily green. The yellow cognitive nodes form a tight cluster at the top, while green and blue symptom nodes form a separate cluster at the bottom. A single red arrow points from Lrn to Ene.

In the Depression-onset group network in the center, the density of connections is significantly higher. Numerous red arrows originate from the yellow Flu node, pointing toward various green and blue symptom nodes like Ene, Ind, and Wor. The blue and green nodes are more tightly interconnected compared to the resilient group.

Navigate back to Image 1:.

